# Spectroscopic investigation of a Co(0001) model catalyst during exposure to H_2_ and CO at near-ambient pressures†

**DOI:** 10.1039/d3cp02739b

**Published:** 2023-07-27

**Authors:** Sabine Wenzel, Dajo Boden, Richard van Lent, Elahe Motaee, Mahesh K. Prabhu, Hamed Achour, Irene M. N. Groot

**Affiliations:** a Leiden Institute of Chemistry, Einsteinweg 55 2333 CC Leiden The Netherlands i.m.n.groot@lic.leidenuniv.nl +31 71 527 7361

## Abstract

Cobalt catalysts, although already used industrially for Fischer–Tropsch synthesis, are prone to a number of deactivation mechanisms such as oxidation of the active phase, and the deposition of carbon and reaction products. We have performed near-ambient-pressure X-ray photoelectron spectroscopy on Co(0001) model catalysts during exposure to gases relevant to Fischer–Tropsch synthesis, *i.e.*, CO and H_2_, at 0.25 mbar total pressure. At this pressure, CO seems to be more efficient at keeping the Co(0001) surface metallic than H_2_, which is the opposite behavior as reported in the literature for other pressure ranges. We offer an interpretation of these differences based on the preferred adsorption and dissociation sites of CO and H_2_ compared to the oxidizing agent water (present as impurity in the gas feed and one of the products of the reaction). Additionally, detailed carbon spectra measured at the HIPPIE beamline of MAX IV allow for the distinction of different adsorbed species: CO and CO_*x*_ species are present in correlation to the presence of oxygen on the surface. Carbidic carbon and graphitic carbon can both be removed by hydrogen, whereas adsorbed hydrocarbons possibly poison the surface.

## Abstract 2

Surface oxidation and the adsorption of different carbon species on a cobalt model catalyst were investigated using near-ambient pressure X-ray photoelectron spectroscopy in the mbar range of H_2_, CO, and Fischer–Tropsch-like reaction conditions.

## Graphical abstract

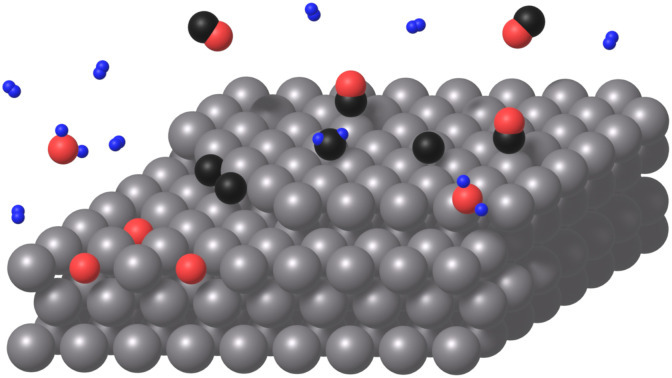

## Introduction

The urgent need for sustainable means of transportation is obvious and reflected in increasingly strict regulations.^1^ For certain applications such as in aviation and maritime shipping, the use of electrical motors will not be feasible in the near future. Here a sustainable solution, which does not require new infrastructure and vehicles, is the use of synthetic Fischer–Tropsch fuels. During Fischer–Tropsch synthesis (FTS) syngas, a mixture of H_2_ and CO is converted into long-chain hydrocarbons.^2^ The syngas can stem from natural gas, oil, or renewable feedstocks such as biomass. Additionally, there is a strong research effort^3,4^ to enable capture and conversion of CO_2_ for the production of syngas, thereby closing the carbon cycle. The synthetic production of fuels will not only reduce CO_2_ emissions compared to conventional fuels, but the resulting fuel is cleaner as well, leading to less CO, NO_*x*_, SO_*x*_, and particulate matter emission than conventional diesel.^5,6^

Fischer–Tropsch catalysis on a large industrial scale has been demonstrated.^7,8^ Typical conditions for so-called low-temperature FTS are 30 bar gas pressure and 200 °C to 240 °C catalyst temperature.^9^ Compared to iron-based catalysts, cobalt supported on various oxide supports shows higher activity and selectivity to linear alkanes.^10–12^ However, cobalt catalysts are prone to multiple deactivation mechanisms, which are being researched intensively.^13,14^ Among these are the deposition of carbon species,^15,16^ sulfur deposition,^17,18^ and the oxidation of the cobalt.^19–22^ Whereas there seems to be a consensus in this literature that cobalt oxide is not active, the ability of cobalt to oxidize under Fischer–Tropsch conditions has been doubted.

As oxidation states as well as adsorption and deposition rates can be highly sensitive to gas pressure and composition, *in situ* spectroscopy studies are indispensable. The use of near-ambient pressure X-ray photoelectron spectroscopy for the study of late transition metal catalysts has been reviewed recently.^23^ Wu *et al.*^24^ have used this technique to determine that, at pressures on the order of 0.1 mbar, CO reduces an oxidized cobalt foil at lower temperatures than hydrogen. In a reaction mixture of 1CO + 1H_2_ the surface stayed metallic at temperatures above 225 °C suggesting that an oxidation of the foil by (background or product) water does not take place under these conditions. Additionally, they have observed carbide formation from CO at temperatures as low as 57 °C. However, it has been shown that the oxidation behavior of cobalt can depend significantly on the crystallinity, structure, and support of the cobalt sample used.^25,26^ This suggests that studies on single crystals are a small but relevant contribution to completely understanding the oxidation behavior of cobalt. Kizilkaya *et al.*^27^ find that H_2_ more readily removes oxygen from Co(0001) than CO. Although this suggests the opposite behavior of Co(0001) compared to Co foil,^24^ the significantly different pressure regime of 10^−5^ mbar used in ref. 27 compared to the mbar range used in ref. 24 could be responsible as well. In the current study, we combine the use of a single crystal with the near-ambient pressure approach in order to investigate the role of background water for the oxidation of Co(0001) in 0.25 mbar H_2_, and compare this to the behavior in 0.25 mbar CO.

The adsorption of reactants, products, and poisons on Co(0001) has been investigated extensively in ultra-high vacuum (UHV) studies.^28,29^ Recently, Chai *et al.* have conducted a near-ambient pressure study on Co(0001) using a lab X-ray source^30^ revealing coverages of CO and other carbon species. With the use of synchrotron radiation, we measured high-resolution spectra on Co(0001), which allow for the distinction of multiple carbon and hydrocarbon species. We provide the amount of surface area covered by these species in H_2_, CO, and mixtures with a H_2_-to-CO-ratio of 2 : 1 and 4 : 1, all at a total pressure of 0.25 mbar and different temperatures ranging from 120 °C to 300 °C. The surface is reduced in H_2_ at 220 °C before introducing the mixtures.

## Experimental

Some preliminary measurements (see ESI†) were performed at beamline 9.3.2 at the Advanced Light Source, Berkeley, US. All measurements presented in the main text were performed at the HIPPIE beamline^31^ of Max IV Laboratory, Lund. A high-pressure cell (similar to the one described in ref. 32) was used here with H_2_ 4.7 (99.997% purity) and CO 3.7 (99.97% purity). Filters are in place to remove impurities such as nickel carbonyls. The H_2_ and CO are expected to have a respective water content of ≤3 ppm and <5 ppm, such that an estimated partial pressure of 10^−6^ mbar water could be expected in the total gas pressures investigated here. Due to the design of the gas system, this was not quantifiable by mass spectrometry. However, a water-induced effect could clearly be observed when hydrogen was used. Therefore, a liquid nitrogen trap was used on the hydrogen line for most of the measurements presented here (labeled as ‘dried hydrogen’). The measurements labeled ‘wet hydrogen’ used the gas as-is from the bottle.

The Co(0001) single crystals were purchased from SPL. They were prepared by cycles of argon ion sputtering with 1 kV acceleration voltage in 1 × 10^−5^ mbar Ar resulting in a sample current of 0.28 μA mm^−2^ for 5 min to 10 min and subsequent annealing to 590 K in UHV for 15 min to 30 min. The annealing temperature is limited by the phase transition from an hcp to an fcc crystal structure.^33^ To additionally prevent contaminants in the bulk from reaching the surface the annealing temperature is 5 °C to 10 °C lower in the last cleaning cycle and the sample is kept at maximally 305 °C during all experiments.

To minimize the influence of beam-induced deposition of carbon species (see ESI†) on the results, a fresh position on the sample is chosen for every set of spectra. Hereby, the carbon spectrum is measured first and two consecutive sweeps are compared to exclude any changes to the surface on the time scale of the measurement.

To allow for quantitative comparison, all spectra are measured at an electron kinetic energy of 200 eV, which corresponds to a probing depth of two to three layers into the cobalt crystal (as estimated from the inelastic mean free path calculated with the help of the NIST Database 71 Version 2.1^34^). The binding energy axes of all oxygen, carbon, and sulfur spectra were calibrated according to the metallic Co 3p_3/2_ peak at 59.3 eV^35^ measured after every change of the photon energy and fitted with a Lorentzian quick fit in Igor Pro 6.37 (which was tested to be in agreement with the peak position determined from a detailed fit in CasaXPS). All further peak fits are done in CasaXPS 2.3.19^36^ using a Shirley background and while fixing the distances between all peaks as well as their full width half maximum.

High-resolution O 1s and C 1s spectra were recorded while maximizing the signal by switching to the largest X-ray entrance slit possible for every spectrum. For quantitative comparison of different O 1s spectra, an additional spectrum was measured at the same slit that the corresponding Co 2p spectrum was measured with (generally a small slit to protect the detector from the strong cobalt signal). The area under this spectrum is then used to normalize the area under the high-resolution spectrum, thereby removing the difference in slit setting. Last, the areas under the Co 2p, high-resolution C 1s, and O 1s spectra are normalized such that the total area under the Co 2p peaks (or a 2nd harmonic contribution of the Co 2p peaks) is the same for different measurement conditions.

Detailed fitting methods of the normalized oxygen and carbon spectra are described in the ESI.† From these, coverages of the different adsorbates on the surface are estimated in comparison with a reference measurement of the saturation coverage of CO in UHV. A more complex analysis taking the attenuation of the different types of adsorbates on the Co signal into account in detail is presented in ref. 37. When the same calibration procedure is used, the simplified method used here leads to results which deviate by maximally 0.08 ML and on average by 12% from the results of the complex analysis. Generally, trends over time and comparison of different values are robust with respect to the exact analysis method as the measurement parameters are kept constant for all measurements. Especially, relative comparisons between different carbon species stem from the same spectrum and are therefore only dependent on the fitting procedure itself. Absolute coverage values, however, appear to mainly depend on the chosen calibration value of the saturation coverage, which is well-known from literature. The presence of other adsorbates and the overall cleanliness and flatness of the sample could influence the measurement used for calibration. By comparing the O coverage estimated for multiple as-prepared samples measured at the same conditions (in UHV at 220 °C), we can generously estimate deviations of up to 30%, which we present as error bars for the absolute values of the estimated coverages. For species with low coverages, peak fitting becomes less reliable such that an absolute minimum error of 0.005 ML is shown.

## Results and discussion

### Oxidation of Co(0001) in hydrogen

**Fig. 1:**
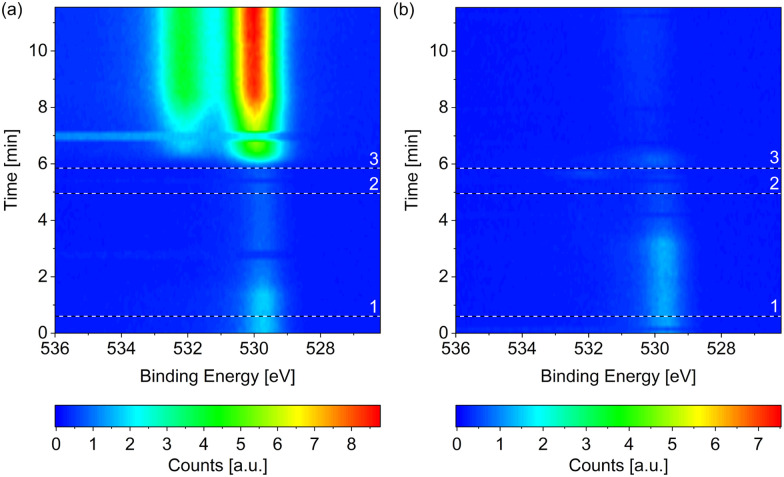
O 1s spectra measured on Co(0001) over time while introducing (a) wet and (b) dried (by a liquid nitrogen trap) H_2_, respectively, both at 220 °C. The black and white reference lines mark (1) the closing of the near-ambient pressure cell, (2) the enabling of the mass flow controller, and (3) the start of the flow of H_2_. All scans in each map were normalized to have the same background level. In (a) two consecutive scans at around 7 min as well as around 3 min do not agree with the scans before and after. Such faulty scans can be caused by mechanical movements on the machine, for example when closing the cell door, or when the detector does not move to the correct energy range in time (meaning that the delay time between different peaks is not chosen long enough). In (b) such faulty scans might be present at 0 min and around 6 min. Both measurements are performed under the same X-ray exposure to exclude beam effects.

Fig. 1 compares the change in the O 1s spectrum while introducing (a) wet (without liquid nitrogen trap) and (b) dried (with liquid nitrogen trap) hydrogen, respectively, both at 220 °C surface temperature. Both samples show some adsorbed oxygen at 529.7 eV when starting from UHV (*t* = 0). As shown in detail in the ESI,† between 0.07 ML and 0.11 ML of adsorbed oxygen is present on the as-prepared samples, which are, however, metallic. A number of as-prepared Co(0001) surfaces in UHV were compared, which results in an average oxidized contribution in the Co 3p or Co 2p peaks of merely 3% of the total depth of cobalt probed. As water is a common background gas in UHV, the oxygen is likely formed on the as-prepared surface by water dissociation. Water has been shown to lead to adsorbed O, which is stable until 350 °C on Co(0001),^38^ and hence cannot be removed without going through the hcp-to-fcc phase transition.

When introducing the wet hydrogen, the oxygen signal strongly increases, whereas the dried hydrogen removes the adsorbed oxygen from the as-prepared sample. It can already be (partially) removed before actually starting the flow of 3.8 ml min^−1^ H_2_ (at the time of reference line 3 in Fig. 1(b)). Due to a step-wise procedure necessary to activate the high-pressure cell and to start the flow, the exact gas composition cannot be known at every point in time between reference lines 1 and 3. This could also explain why it appears that the adsorbed oxygen on the as-prepared sample is (partially) removed in Fig. 1(a) before it strongly increases in the flow of wet H_2_.

**Fig. 2:**
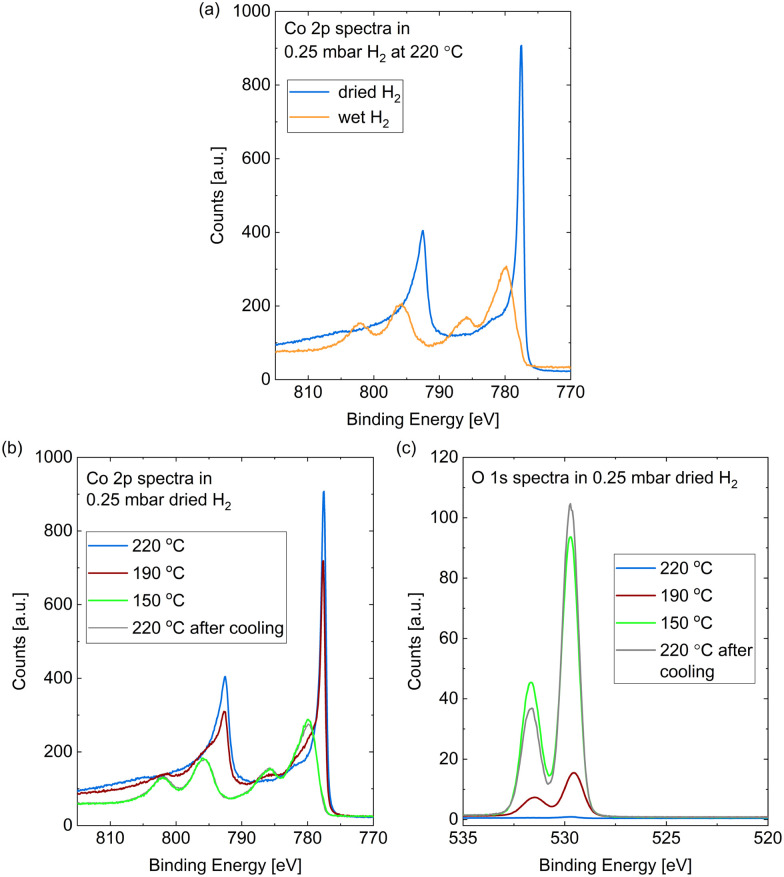
(a) Co 2p spectra measured *in situ* on Co(0001) after roughly 30 min in 0.25 mbar dried H_2_ (blue) compared to after roughly 10 min in wet H_2_ (orange), both introduced at 220 °C. (b) Co 2p and (c) O 1s spectra measured on Co(0001) in 0.25 mbar dried H_2_ at 220 °C, 190 °C, and 150 °C, as well as after subsequent heating to 220 °C. For quantitative comparison of the O 1s spectra they are calibrated for the used slit setting and the Co 2p peak area as explained above.

The Co 2p peaks displayed in Fig. 2(a) suggest that the probed depth of the cobalt sample is almost completely oxidized by the wet hydrogen. 98% of the peak area is identified as oxidized cobalt in a detailed fit (see fitting procedure and identification of the oxide as CoO in the ESI†).

The strong contribution at around 530 eV in the O 1s spectrum in Fig. 1(a) can thus be identified as mainly stemming from CoO instead of adsorbed atomic oxygen. However, a second, higher binding-energy contribution is clearly visible in Fig. 1(a). As there is no measurable carbon signal at the time of the last spectrum of the map (data not shown), any background CO can be excluded as the origin of this peak. Multiple peaks could be stemming from the CoO^39^ although the distance between the peaks (measured as 2.1 eV as shown in the ESI†) does not fully agree with the literature value, which could hint at a somewhat different oxidation state of the oxygen in the surface region or in the region between oxide layer and metallic bulk cobalt. Another possibility would be that the higher binding-energy peak stems from molecularly adsorbed water, which is detected at varying binding energies depending on co-adsorbates, the oxidation state of the cobalt, and the amount of water.^24,38,40^ Water is expected to only adsorb dissociatively on clean Co(0001) as well as on O(ad)/Co(0001) in UHV at the temperature investigated here.^38,41^ On the other hand, CoO has been shown to be inactive for water dissociation,^38^ such that the presence of molecularly adsorbed water on top of the oxidized cobalt in wet hydrogen cannot be excluded.

In comparison to the oxidation in wet hydrogen, the surface stays mostly metallic in the dried hydrogen (see Fig. 1(b) and Fig. 2(a)). The detailed fit shows 9% oxidized contribution in the Co 2p peaks, which is not more than the maximum measured on an as-prepared sample in UHV.

However, the surface does oxidize in the dried hydrogen as well upon reduction of the temperature. Fig. 2(b) shows the Co 2p spectra measured in 0.25 mbar dried H_2_ under cooling down from 220 °C to 190 °C and 150 °C, as well as under subsequently increasing the temperature to 220 °C again. The sample was held at each of the temperatures for about 1 h and the effects described in the following were checked to be constant in this time frame. At 190 °C a partial oxidation is clearly visible in the Co 2p spectrum (red curve). A detailed fit identifies 38% of the probed depth measured as oxidized cobalt. A full oxidation of the probed depth is measured at 150 °C (green curve). Fig. 2(b) additionally displays the Co 2p spectrum after increasing the temperature of the oxidized surface from 150 °C to 220 °C (grey curve), where it is not reduced again. Therefore, we can identify the reduction as kinetically limited in the time frame investigated here.

**Fig. 3:**
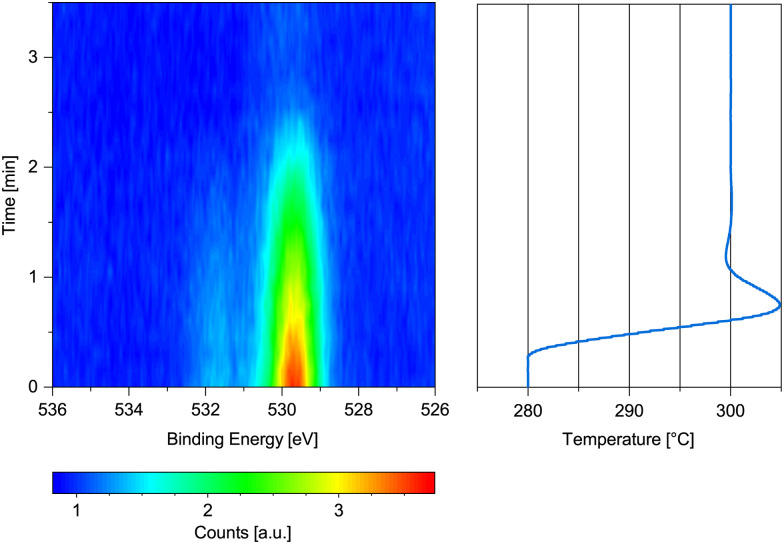
O 1s spectrum measured on previously oxidized Co(0001) in 0.25 mbar dried H_2_ while increasing the temperature from 280 °C to 300 °C with the corresponding temperature graph.

Fig. 2(c) shows the corresponding O 1s spectra during the cooling and the subsequent heating, which confirm the oxidation as both contributions increase with increasing oxidation in the Co 2p spectra. However, the area ratio of the two O 1s contributions does not stay strictly the same. As the higher binding-energy contribution does not vanish when the whole probing depth is oxidized (for example at 150 °C), we can exclude an interface oxide between the oxide layer and the metallic cobalt as its origin. However, when increasing the temperature to 220 °C the area under the higher binding-energy peak decreases. It is more strongly reduced at the time of the first oxygen spectrum (*t* = 0) in the map in Fig. 3, which was measured at 280 °C, while the lower binding-energy contribution is still clearly visible. Temperature-programmed desorption measurements by Xu *et al.*^38^ suggest that molecular water could start to desorb from an oxidized surface between roughly 80 °C and 130 °C. However, they find the desorption temperature to increase with increasing amount of oxidation. It is possible that our surface is more oxidized than the surface in the studies of Xu *et al.*, leading to a higher desorption temperature (between 150 °C and at 220 °C as suggested by the decrease in the peak area). Another possibility for the origin of the high binding-energy peak are less-oxidized cobalt species, which might be reduced more easily.

Fig. 3 shows the change in the O 1s signal *versus* temperature. Upon increasing the temperature, the sample is reduced within 2 min above 280 °C. A 90% metallic contribution was subsequently measured in the Co 2p spectrum at 300 °C. However, the increase of the temperature from 220 °C to 280 °C took place within only 15 min, leaving the possibility that a lower temperature might have been sufficient for reduction over the course of hours. The rather fast onset of the reduction upon increase of the temperature while already continuously measuring the oxygen spectrum in Fig. 3 for some time excludes a beam-induced reduction, however.

Generally, hydrogen shows a low sticking probability on cobalt (see ref. 42 and references therein). The hydrogen that does adsorb is more stable on the terraces of Co(0001) than on the steps and defects,^42,43^ such that these lower-coordinated Co sites could be free for dissociative H_2_O adsorption as soon as the temperature is low enough in the specific H_2_O partial pressure. DFT calculations by Ma *et al.*^44^ suggest that the adsorption of H_2_O is significantly easier on the steps than on the terraces, whereas the dissociation is only slightly enhanced in comparison. Once some H_2_O has dissociated, the resulting adsorbed O can strongly promote further H_2_O dissociation on the terrace^38,45^ and even more so on the steps.^44^ The oxidized CoO/Co(0001) terraces are not active for further water dissociation,^38^ such that a limited growth of only one layer of oxide could be expected. However, to our knowledge there are no similar studies regarding the water dissociation on the steps and defects of oxidized cobalt. As the measurements presented here probe between two and three layers of the single crystal, which appear fully oxidized, we cannot directly confirm or exclude a limited growth.

Under vacuum pressures, where hydrogen has been shown to remove adsorbed oxygen from Co(0001) from roughly 180 °C onwards^27^ and no oxidation caused by H_2_ has been reported, the significantly lower background water pressure could be insufficient to enable the water adsorption and dissociation for oxidation on the typical time scale of an experiment. The same study finds that a higher temperature is needed for H_2_ to remove adsorbed oxygen from defective Co(0001) compared to the Co(0001) terraces, which confirms that it is less effective at the low-coordinated sites.

In a pressure of 0.2 mbar H_2_, Papaefthimiou *et al.*^26^ have reduced an oxidized Co(0001) surface at about 250 °C, which is the same range as our result taking the usual inaccuracies of temperature measurements into account. Additionally, our measurements agree with the results of Wu *et al.*^24^ on polycrystalline cobalt foil. In roughly 0.13 mbar (100 mTorr) H_2_ they observe oxidation below 185 °C and the reduction between 200 °C and 290 °C indicating that the difference in structure between the single-crystalline and the polycrystalline sample might not have a significant influence on the oxidation behavior in this pressure range of hydrogen.

### Metallic Co(0001) in CO

**Fig. 4:**
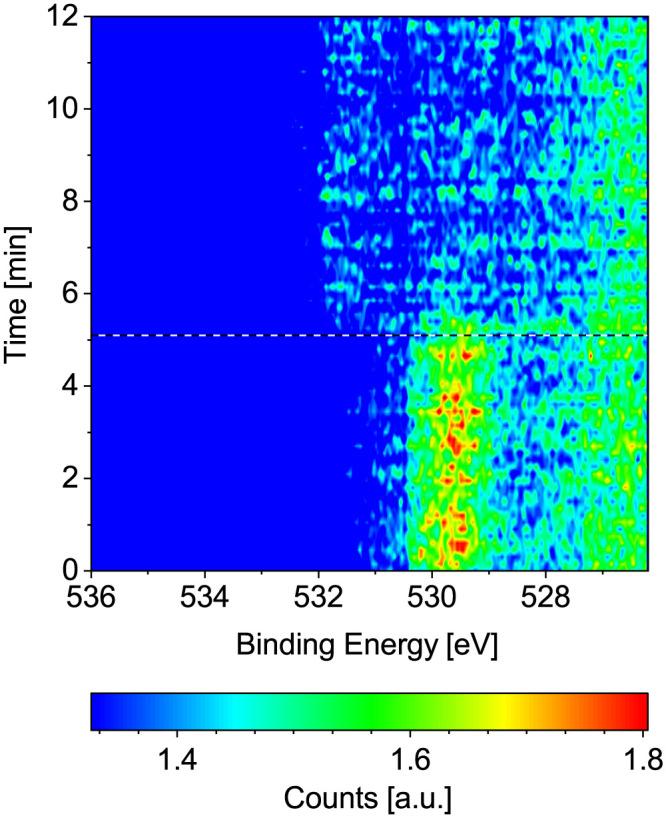
O 1s spectra measured on Co(0001) over time while introducing CO at 220 °C. The black and white reference line marks the start of the flow of 1.9 ml min^−1^ CO.

Fig. 4 displays the change of the O 1s signal on Co(0001) while introducing CO into the near-ambient-pressure cell at 220 °C surface temperature. The adsorbed oxygen on the as-prepared sample is efficiently removed as soon as the CO is introduced (see horizontal reference line). At the same time, the adsorbed CO signal appears at higher binding energy, albeit not yet to a strong extent compared to the adsorbed O on the as-prepared sample.

The Co 2p signals displayed in Fig. S4 in the ESI† confirm that the cobalt stays mainly metallic in 0.25 mbar CO at 220 °C (0.5% oxidized contribution in the fit). This is still the case when decreasing the temperature to 120 °C (4% oxidized contribution in the fit). Thus, although the CO contains a similar amount of water as the hydrogen and is not additionally dried, it is significantly more efficient at keeping the Co(0001) metallic compared to dried H_2_ at the same total pressure. This is especially surprising as the CO is expected to dissociate on defect sites starting from around 60 °C onwards.^29^ Thus, at least part of the resulting adsorbed atomic oxygen desorbs as CO_2_ without oxidizing the cobalt.

These results are in agreement with the measurements of Wu *et al.*^24^ on cobalt foil, which stays metallic even at room temperature in roughly 0.13 mbar CO. Additionally, they measure the reduction of CoO/Co(0001) by CO between 150 °C and 200 °C. Compared to the metallic Co(0001) in CO at 120 °C observed here, this could again indicate a kinetic limitation for the reduction by CO although deviations in the detected temperature between our measurements and the literature cannot be excluded. The results by Wu *et al.* do show a more efficient reduction by CO compared to H_2_ at the same total pressure as observed here.

Co_3_O_4_ powder has also been shown to start reducing in 0.15 mbar CO from 150 °C on, at lower temperatures than in 0.4 mbar H_2_.^46^ In UHV studies, Kizilkaya *et al.*^27^ observe the opposite behavior as 2 × 10^−5^ mbar H_2_ removes adsorbed oxygen from Co(0001) at 177 °C, whereas CO in the same pressure range does not remove it even at 357 °C. Although CO shows high sticking probabilities on metal surfaces, a coverage of at most 1/3 ML of CO and mainly on the top sites can be expected under these conditions (estimated from Fig. 8 in ref. 28). Based on the same reference, a coverage between 1/3 ML and 0.5 ML can be expected on the top as well as the hollow sites in all pressure and temperature conditions studied here (as well as industrial FTS conditions). As the top sites would be the most stable position for H_2_O as well,^45,47^ a specific minimal coverage with CO, thus minimal CO pressure at a specific temperature, could be needed for sufficient site blocking to prevent the oxidation. Combined TPD and DFT studies by Jiawei *et al.*^41^ suggest that the adsorption of CO is significantly stronger than the adsorption of H_2_ O such that the CO could adsorb preferentially when both are present in the gas phase.

As water preferentially adsorbs on the steps compared to on the Co(0001) terrace,^44^ the steps might be more relevant than the different terrace sites here. In contrast to H_2_, CO shows a comparable adsorption on stepped surfaces as on Co(0001).^48^ Thus, the blocking of step sites by the CO at the investigated pressures is possible. At the same time, the dissociation of CO has been shown to be facilitated at the steps of Co(0001) compared to the terrace^29^ and to not be hindered by an increasing CO coverage.^49^ The dissociation could lead to additional blocking of the step edges by the resulting atomic carbon (which we discuss in the following section).

### Adsorbed species on Co(0001) in CO

**Fig. 5:**
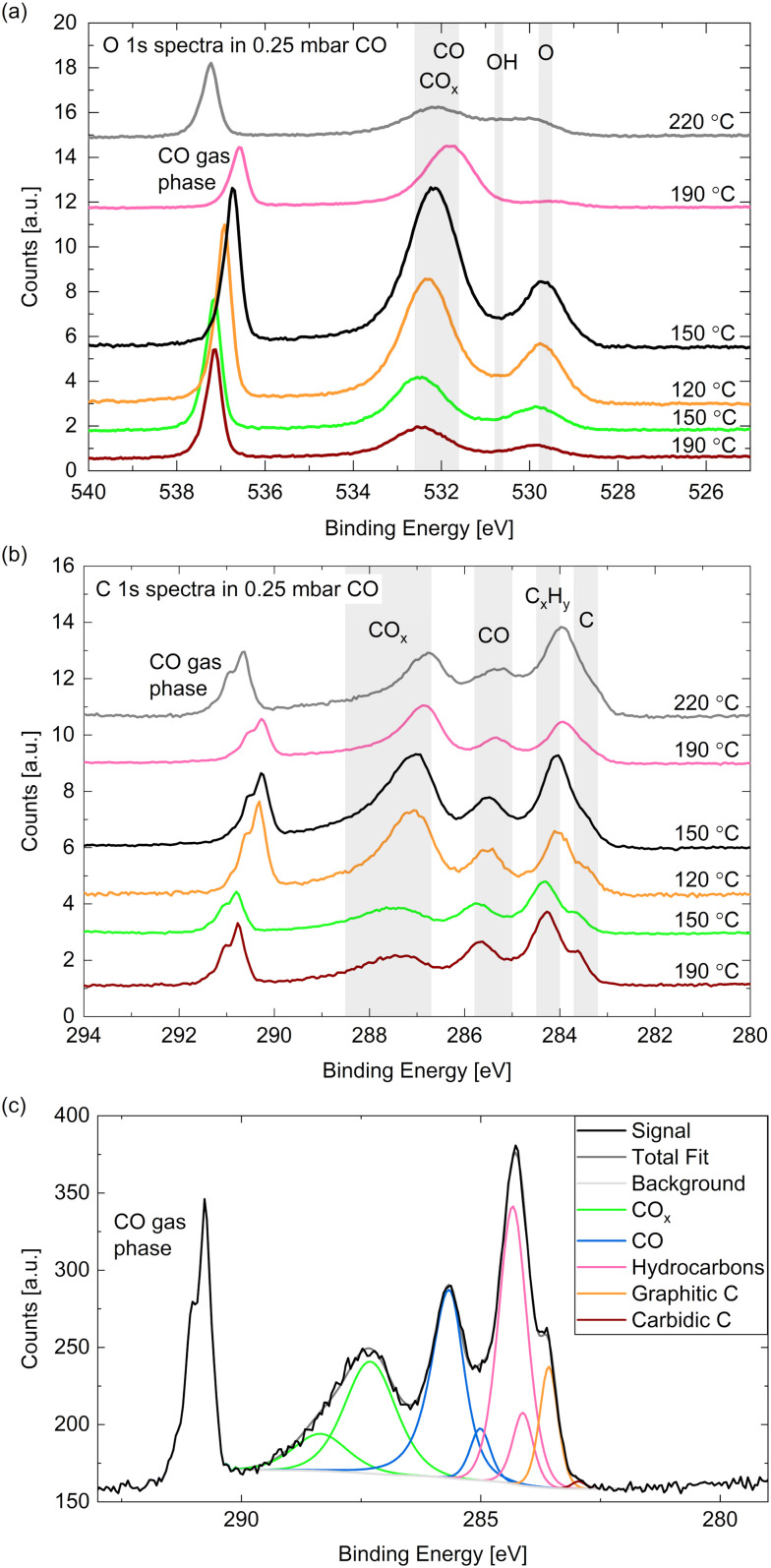
(a) O 1s and (b) C 1s spectra measured on Co(0001) in 0.25 mbar CO at different temperatures. For quantitative comparison the spectra are calibrated to the Co 2p peak area as explained above. (c) C 1s area measured on Co(0001) in 0.25 mbar CO at 190 °C surface temperature showing the peaks necessary for a satisfactory fit.

Fig. 5(a) and (b) show high-resolution O 1s and C 1s spectra measured in 0.25 mbar CO. The temperature was decreased from 190 °C to 120 °C and subsequently increased to 220 °C (from bottom to top in the figure). In comparison with relative peak positions presented in the literature,^24,40,50^ one can identify the binding energy regions where adsorbed CO_*x*_ (identification see below), CO, hydroxyls, and oxygen (or cobalt oxide) can be found as marked with the grey areas in Fig. 5(a). In the same manner we identify – in order of decreasing binding energy – gas phase CO, adsorbed CO_*x*_ (details see below), adsorbed CO, hydrocarbons C_*x*_H_*y*_, and carbon atoms in the C 1s spectra (see Fig. 5(b)). The detailed fitting procedure used for all C 1s spectra is explained in the ESI† and shown in the example in Fig. 5(c). It is used to estimate the coverages of the adsorbed species mentioned in the following.

The gas phase peak appears at a lower binding energy at lower temperatures, which indicates an increase in the work function of the surface, in agreement with measurements on polycrystalline cobalt.^24^ The deviations in the area under the gas phase peak could indicate that the exact CO pressure between the sample and the aperture decreases with the sample temperature (as observed in ref. 30). Although we have found the cobalt surface to stay metallic under these conditions (see above), an oxygen peak around 529.7 eV is visible, which thus needs to be identified as adsorbed atomic oxygen. This contribution is clearly increased at lower temperatures with an estimated overage of up to 0.08 ML.

The CO contribution in the O 1s signal appears significantly larger at lower temperatures as well. However, the corresponding peak (between 531 eV and 533 eV) seems to shift between the different temperatures, which suggests that two different species are contributing to it. This separation is more distinct in the C 1s spectra where two peaks are clearly visible between 288 eV and 285 eV. As the higher binding energy contribution appears simultaneously with an increased oxygen signal it might stem from a CO species co-adsorbed with O or a CO_*x*_ species. The presence of O has been shown theoretically^51^ and experimentally^29^ to decrease the probability of CO adsorption and dissociation while CO_2_ formation is favored (observed above 10^−4^ mbar CO). In UHV studies CO_2_ is observed to desorb from the surface from around 230 °C on,^29^ which suggests that it could be present at the conditions investigated here. To our knowledge, there is no evidence in the literature regarding the presence of CO_*x*_ species with *x* > 2 on Co(0001). Additionally, it is possible that carbonyl species Co(CO)_*x*_ appear at these binding energies.^52^ Based on comparison to other catalytic materials, it has been previously suggested that carbonyls with *x* = 1–4 could be present on Co(0001) under CO hydrogenation conditions.^53^

**Fig. 6:**
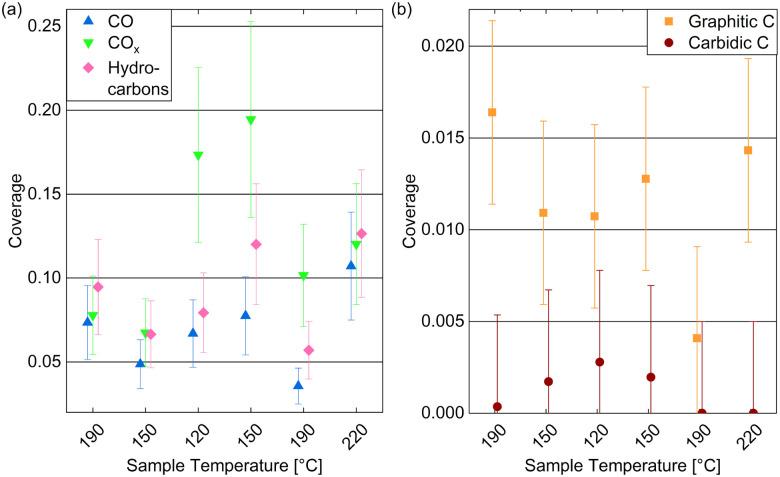
Estimated coverages of (a) adsorbed CO, CO_*x*_, and hydrocarbons, as well as (b) graphitic compared to carbidic carbon on Co(0001) in 0.25 mbar CO at temperatures decreasing from 220 °C to 120 °C and subsequently increasing to 220 °C. The coverages are estimated from the peak areas of detailed fits. Error bars indicate the estimated accuracy of the absolute coverage values due to the calibration method while relative comparisons of the different measurements are expected to be more reliable (see Methods section).

Fig. 6(a) displays the estimated coverages of CO_*x*_, CO, and hydrocarbons. The CO coverage in 0.25 mbar CO fluctuates between 0.04 ML and 0.11 ML without a clear trend with the temperature, whereas the CO_*x*_ species increases from 0.08 ML to 0.19 ML when decreasing the temperature, which is likely related to the increased amount of O available. In the detailed fitting procedure (see ESI† and overview in Fig. 5(c)) two CO_*x*_ peaks as well as two CO peaks can be distinguished. Most of the CO is found in the first higher binding-energy peak at all investigated temperatures (between 83% and 88% of the total CO contribution), which can thus be identified as the more stable adsorption site. Compared to the phase diagram for CO adsorption by Weststrate *et al.* (Fig. 8 in ref. 28), this is likely the top site, whereas the second observed one can be attributed to the hollow site. For the CO_*x*_ species, however, the lower binding-energy peak is the dominant one (and thus likely the more stable site) as it accounts for between 71% and 83% of the CO_*x*_. The change of the amount of adsorbed hydrocarbons with temperature (see Fig. 6(a)) appears somewhat correlated to the amount of adsorbed CO. If all hydrocarbons stemmed from the CO bottle, more adsorbed hydrocarbons could be expected after decreasing the temperature to 150 °C. On the other hand, a larger hydrocarbon coverage at higher temperatures from 190 °C on could be expected if the hydrocarbons are formed through reaction of the adsorbed CO with background hydrogen. Therefore, a clear identification as a reaction product cannot be made on the basis of these measurements. The absolute atomic carbon coverage is not significant with values between 0.01 ML and 0.02 ML, such that the change with temperature might fall in the inaccuracy of the estimated coverages. Although CO dissociation, which can be expected from 60 °C onwards,^29^ could leave carbon behind, the amount of carbon is in the same order of magnitude as on the as-prepared sample in UHV at 220 °C. Thus, it cannot be related to CO dissociation unambiguously. This is in contrast to results by Chai *et al.*^30^ who find significantly more carbon in 0.5 mbar CO at the same temperature, which could be related to the larger pressure or possibly a longer waiting time in the same conditions.

In the detailed fit we distinguish graphitic C from carbidic C (identified based on ref. 54 and 55). The resulting estimated coverages are displayed in Fig. 6(b). As opposed to in UHV, the carbon is mainly present as graphitic carbon, whereas a smaller amount of carbidic carbon that appears when decreasing the temperature to 150 °C is not visible anymore after increasing it to 190 °C again. The carbidic carbon can diffuse into the bulk, although we have observed that only at higher temperatures in UHV. It is more likely that carbon is removed by reaction with background H_2_ from 190 °C on. Interestingly, the graphitic carbon seems to be present in larger amounts at higher temperatures. This could be due to a higher rate of CO dissociation or also be related to the decrease in carbidic carbon at those temperatures.

### Adsorbed species on Co(0001) in hydrogen

**Fig. 7:**
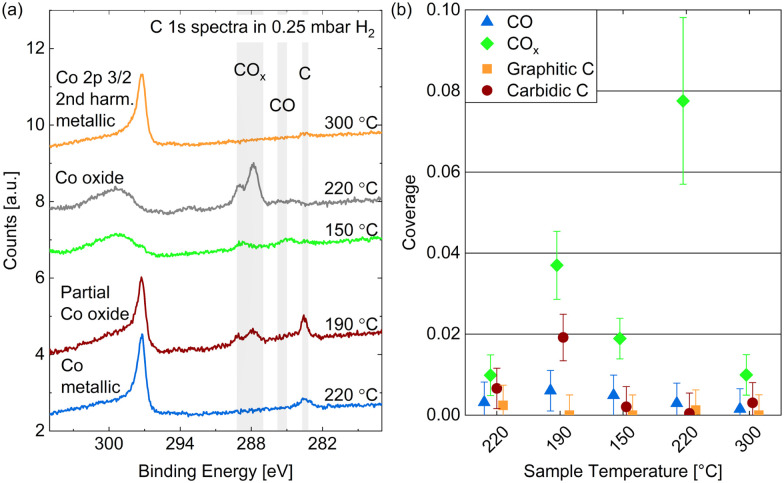
(a) C 1s spectra measured on Co(0001) in 0.25 mbar dried H_2_ at different temperatures. For quantitative comparison the spectra are calibrated using the Co 2p_3/2_ 2nd harmonic component visible at the high binding-energy side of the carbon spectra. (b) Estimated coverages from detailed fits distinguishing carbidic from graphitic C and CO from CO_*x*_ as a function of the surface temperature. Error bars indicate the estimated accuracy of the absolute coverage values (see Methods section).

Fig. 7(a) shows the C 1s spectra measured in 0.25 mbar dried hydrogen at the same temperatures as studied above: cooling down from 220 °C to 150 °C and subsequently heating to 220 °C and 300 °C (from bottom to top in the figure). The Co 2p_3/2_ component caused by second harmonic photons confirms the oxidation behavior discussed above. As the amount of carbon species found is small, we provide estimated coverages from detailed fits in Fig. 7(b) which are, however, prone to significant inaccuracies.

The amount of carbon present on the surface at 220 °C is on the same order of magnitude as in UHV and in CO. However, as opposed to in CO, mainly carbidic carbon is detected at all temperatures, which suggests that the formation of graphitic carbon is suppressed by the presence of hydrogen. This can be expected, as the adsorption of and reaction with hydrogen prevents the carbidic carbon forming bonds with other carbon atoms, and potentially forms hydrocarbon products, mainly methane at the investigated conditions,^50^ which desorbs from the surface. When decreasing the temperature from 220 °C to 190 °C the amount of carbon clearly increases, which agrees with the behavior of the carbidic carbon in 0.25 mbar CO and is thus likely related to an increased adsorption and dissociation of background CO. This trend does not continue when decreasing the temperature further to 150 °C and for the subsequent measurement at 220 °C, which is likely related to the oxidation of the surface. However, the metallic surface at 300 °C does not show significant carbon coverages again. Overall, the removal of carbon seems to start between 190 °C and 220 °C on the Co(0001) surface at a slightly higher temperature compared to around 150 °C observed on cobalt foil.^24^

The presence of some adsorbed CO is generally not surprising as there is a CO background in the UHV chamber as well as in the gas lines after previous uses of CO in the cell. However, no clear trend with temperature in H_2_ can be recognized in Fig. 7(b) except that no CO is adsorbed on the metallic Co(0001) at 300 °C. The presence of CO_*x*_, however, is clearly correlated to the (partial) oxidation of the cobalt at 190 °C, 150 °C, and the subsequent 220 °C measurement. It is not surprising that the presence of a large amount of oxygen would allow for the formation of CO_*x*_. Hereby, a higher temperature appears to aid the formation as the coverage decreases slightly when cooling down to 150 °C and increases from 0.003 ML to 0.07 ML when subsequently heating the oxidized surface to 220 °C.

### Adsorbed species on metallic Co(0001) in FTS-like mixtures

**Fig. 8:**
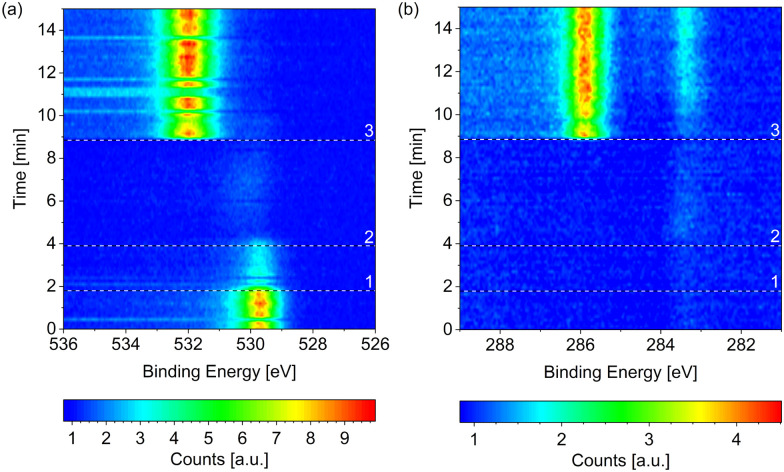
Change of the (a) O 1s and (b) C 1s signals measured on Co(0001) over time while introducing the reaction mixture of 4H_2_ + 1CO at 220 °C. The three black and white reference lines mark (1) the moment the mass flow controllers are connected to the cell, (2) the start of the flow of 4.4 ml min^−1^ H_2_, and (3) the start of the flow of 1.1 ml min^−1^ CO. All consecutive spectra have been normalized to the background level at low binding energy. Some faulty measurements caused by an insufficient delay between the different scans are visible as horizontal lines in (a).

Starting from the as-prepared Co(0001) in UHV, the reaction mixture was introduced by first starting the flow of dried H_2_ at 220 °C surface temperature and, once all adsorbed oxygen has been removed, starting the additional flow of CO (at 1/2 or 1/4 of the H_2_ flow, respectively). Finally, the total pressure is increased to 0.25 mbar. This process can be seen in Fig. 8(a) in the form of the removal of O and the subsequent adsorption of CO at higher binding energy. Right after the start of the CO flow, the adsorbed CO as well as carbon (at lower binding energy) can be seen in the change of the C 1s signal in Fig. 8(b). Additionally, a small amount of adsorbed OH and some carbon or hydrocarbon species could be suspected in H_2_ (between reference line 2 and reference line 3). However, these are not significant compared to the background level.

As the cobalt stayed metallic in 0.25 mbar dried H_2_ as well as 0.25 mbar CO (see above) at 220 °C, it could be expected that it also stays metallic in a mixture of the two gases at the same total pressure and temperature. This is confirmed by an oxidized contribution to the Co 2p fit of at most 3% during up to 3 h in a 4 : 1- or a 2 : 1-ratio of H_2_ to CO at 220 °C. After a subsequent increase to 250 °C the same value is measured. From high-resolution O 1s spectra it is estimated that the coverage of adsorbed atomic oxygen is not significant either during the reaction with at most 0.01 ML for both gas ratios at 220 °C and only slightly lower with a maximum of 0.008 ML at 250 °C. Lower temperatures were not investigated in the reaction mixtures here. However, the observation that the surface is metallic at 220 °C is in rough agreement with the reduction of oxidized cobalt foil seen from 225 °C on in 0.1 mbar 1H_2_ + 1CO.^24^

**Fig. 9:**
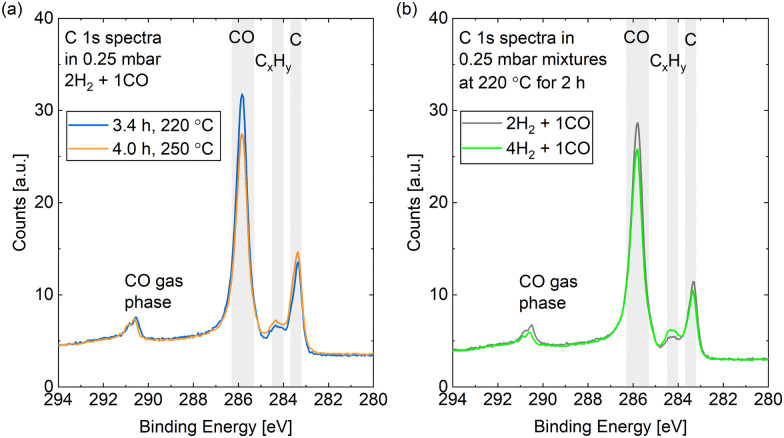
C 1s spectra measured (a) in 0.25 mbar 2H_2_ + 1CO comparing a measurement at 220 °C to a subsequent one at 250 °C and (b) at 220 °C comparing a measurement in 0.25 mbar 2H_2_ + 1CO to one in 0.25 mbar 4H_2_ + 1CO.

**Fig. 10:**
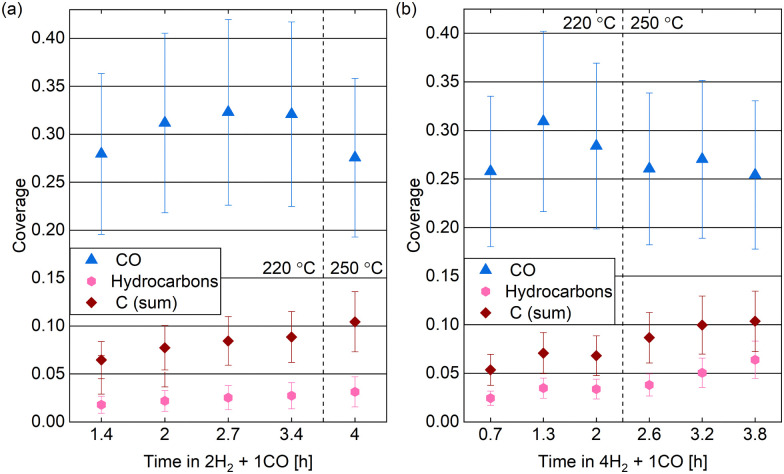
Estimated coverages of CO, hydrocarbons, and carbon (sum of the graphitic and the carbidic carbon) in (a) 0.25 mbar 2H_2_ + 1CO and (b) 0.25 mbar 4H_2_ + 1CO over time at two different temperatures. Error bars indicate the estimated accuracy of the absolute coverage values due to the calibration method while relative comparisons of the different measurements are expected to be more reliable (see Methods section).

Fig. 9(a) compares the C 1s spectra measured in 0.25 mbar 2H_2_ + 1CO before and after increasing the surface temperature from 220 °C to 250 °C. The most prominent difference is that less CO is adsorbed at the higher temperature, which holds for a reaction mixture of 0.25 mbar 4H_2_ + 1CO as well (data not shown). The coverage of CO is estimated to be between 0.25 ML and 0.32 ML for both mixtures without a clear time dependence in the first hours (see Fig. 10). As parts of the surface are covered with other adsorbates, a more useful value is the estimated coverage on the bare part of the surface. This is calculated to be between 0.3 ML and 0.4 ML during exposure to the mixtures. Fig. 9(b) exemplifies that the CO coverage is only slightly higher in 2H_2_ + 1CO than in 4H_2_ + 1CO and thus not strongly dependent on the exact CO pressure in the range measured here. This is confirmed by the fact that the CO coverage on the bare part of the surface in 0.25 mbar CO at 220 °C is comparable as well with 0.3 ML. This value is in agreement with the total CO coverage measured in 1.5 mbar at 220 °C.^30^ The estimated coverage (on the bare part of the surface) in the mixtures is somewhat lower than the coverage of 0.5 ML which is maximally expected during the reaction,^28^ but could still be in agreement taking the accuracy of ± 30% of our absolute coverage values into account. If measured with more accuracy, the difference could indicate a pressure gap between the mbar range and the bar range of pressures.

In the reaction mixtures, between 93% and 97% of the CO is found in the peak associated with the most stable site, which is likely the top site.^28^ Thus, although the absolute coverage in reaction conditions is comparable to in CO only, a somewhat higher fraction of it is on the top site. This might be related to the smaller amount of other adsorbates, which allows for more CO to occupy the preferred position. Chai *et al.*^30^ find higher coverages with other carbon species and only identify around 50% of the CO as adsorbed in the most stable site under reaction conditions.

Additionally, we find a CO_*x*_ coverage estimated to be at most 0.01 ML in reaction conditions, which agrees with the low amount of adsorbed atomic oxygen. This is an order of magnitude lower than in CO at the same temperature, which indicates that H_2_ prevents the CO_*x*_ formation at 220 °C.

The spectra in Fig. 9 show a clear contribution in the area identified as atomic carbon. For a more detailed investigation of the C coverage, Fig. 10(a) and (b) show the change of the estimated total carbon coverage (sum of graphitic and carbidic peak) over time. Although an increasing trend is visible at 220 °C, the C coverage stays between 0.05 ML and 0.08 ML over a time of 3 h at this temperature. In the first measurement in time, the C coverage is already significantly higher than in H_2_ or CO at 220 °C suggesting that it is caused by reaction between the two gases. Fig. 9(b) shows that the carbon deposition is not faster in 2H_2_ + 1CO than in 4H_2_ + 1CO, suggesting that the removal of C is not limited by the exact H_2_ partial pressure in this pressure range. Additionally, there is no clear trend in the C coverage when increasing the temperature to 250 °C (see Fig. 10(a) and (b)) and the further C deposition at this temperature (measured only in 4H_2_ + 1CO) does not display a larger rate than at 220 °C. Thus, any increase in the rate of CO dissociation with increased temperature is compensated by a faster C removal by H_2_. These observations seem to be in contrast to magnetometer studies on an industrial catalyst where the rate of carbide formation, although deemed insignificant in general, was found to decrease with an increased H_2_-to-CO-ratio as well as with increased temperature.^56^ However, a direct comparison of such literature to our experiments is not justified given the use of a single crystal, the lower pressure range, and significantly shorter time frame investigated here. The detailed distinction between the two carbon contributions shows that carbidic and graphitic carbon follow a similar trend in time. With an average of 70% the fraction of carbidic carbon is larger than in CO but smaller than in H_2_, again suggesting that the graphitic carbon is removed by the hydrogen more easily.

Starting out with an absolute value of around 0.02 ML at 220 °C in both reaction mixtures, there are more hydrocarbons adsorbed than in H_2_ but less than in 0.25 mbar CO. However, the absolute hydrocarbon coverage becomes higher in 4H_2_ + 1CO than in 2H_2_ + 1CO, thus scaling inversely with the CO partial pressure. This can for example be seen by comparing the measurement after 2 h at 220 °C and the first measurement at 250 °C for the two different gas ratios in Fig. 10(a) and (b). Thus, it can be excluded that all hydrocarbons stem from the background of the CO gas. Additionally, the hydrocarbon coverage follows roughly the same trend over time as the C coverage including a stronger increase after increasing the temperature indicating that at least part of the hydrocarbons could stem from the same reaction process as the carbon. Mainly methane and other short products can be expected on Co(0001), especially in the mbar pressure range.^50,57,58^ The methane turnover on Co(0001) does increase with increasing H_2_-to-CO-ratio.^59,60^ However, even if it is the main product, methane will likely not stay adsorbed on the Co(0001) surface^61^ and is thus not the species detected by XPS. In contrast, it can be expected that the adsorption strength of the products increases roughly linearly with the chain length^62,63^ and that a chain length of at least 15 carbon atoms is needed to reach a significant coverage on the Co(0001) surface.^64^ The sum of the estimated coverages of all adsorbates stays within 0.36 ML and 0.47 ML for both reaction mixtures over the time of hours tested here. This confirms that the surface is not fully covered with products and excludes any influence of space limitation on the observed coverages. Generally, the deposition of products, although it can poison the catalyst,^13^ can also be considered part of the initiation phase of the catalyst after which the desorption of subsequent products is facilitated.^64^

## Conclusions

Generally, the oxidation behavior of cobalt during FTS depends on a number of factors: the crystal structure of the sample, the water partial pressure, and the H_2_-to-CO-ratio. For the case of Co(0001), CO is more efficient at keeping the cobalt metallic compared to H_2_ at the same total pressure of 0.25 mbar as measured here by *in situ* X-ray photoelectron spectroscopy. We have suggested that this behavior might be explained on the basis of the different adsorption and dissociation sites of H_2_, CO, and H_2_O, respectively. Namely, based on existing literature we expect that CO adsorbs equally well on the steps and vacancies as on the terrace sites^48^ and is therefore able to block the dissociation of water, which most likely occurs at low-coordinated sites.^44^ In comparison, H_2_ is expected to prefer the Co(0001) terrace sites for adsorption^42,43^ and can therefore not prevent the H_2_O dissociation in this pressure range. Analysis of the oxygen spectra suggests that molecularly adsorbed water can collect on top of the oxidized cobalt.

Under FTS-like reaction conditions of 4H_2_ + 1CO and 2H_2_ + 1CO at 220 °C to 250 °C the cobalt surface stays mainly metallic.

Additionally, high-resolution carbon spectra allow for the distinction of a number of adsorbed species. In this way it can be estimated that 70% of the carbon adsorbed on the surface during the reaction at 220 °C is carbidic and the rest graphitic. Although the latter is removed first, both can be removed by hydrogenation at this temperature. To a lesser extent, adsorbed hydrocarbons have been observed as well. Although the adsorbed hydrocarbon species are likely not the main reaction product under the conditions studied here, this adsorption can potentially contribute to poisoning of the active sites. For a clear identification of different types of hydrocarbons as well as the different peaks associated with adsorbed CO and CO_*x*_, respectively, the surface should be investigated in a larger CO pressure range from UHV to mbars.

## Author contributions

S. Wenzel: main investigation, formal analysis, visualization, writing. D. Boden: investigation. R. van Lent: investigation. E. Motaee: investigation. M. K. Prabhu: investigation. H. Achour: investigation. I. M. N. Groot: conceptualization, supervision, funding acquisition.

## Conflicts of interest

There are no conflicts to declare.

## Supplementary Material

CP-025-D3CP02739B-s001
