# Supplementary material for: Spectroscopic investigation of a Co(0001) model catalyst during exposure to H2 and CO at near-ambient pressures
Source: Phys Chem Chem Phys. 2023 Jul 27;25(37):25094–104. doi: 10.1039/d3cp02739b (PMC10528786; doi:10.1039/d3cp02739b)
Supplement: CP-025-D3CP02739B-s001 [file CP-025-D3CP02739B-s001.pdf]

Electronic Supplementary Material (ESI) for Physical Chemistry Chemical Physics.
This journal is © the Owner Societies 2023

Supplementary Information to "Spectroscopic investigation of a Co(0001) model catalyst during exposure to H_2 and CO at near-ambient pressures"

Sabine Wenzel,[a,b] Dajo Boden,[a] Richard van Lent,[a] Elahe Motaee,[a] Mahesh K. Prabhu,[a] Hamed Achour,[a] and Irene M.N. Groot*[a]

As-prepared, metallic Co(0001) in UHV

Figure S1 shows the Co 2p, O 1s, and C 1s spectra of the as-prepared sample in UHV comparing a measurement at 20 °C to one at 220 °C. Both measurements have been taken after CO has been used in the high-pressure cell inside the analysis chamber, which increased the base pressure from the 10^{-10} mbar to the 10^{-9} mbar range compared to the freshly-baked analysis chamber.

In comparison with relative peak positions given in literature[1–3], we can identify the binding energy regions where adsorbed oxygen (or cobalt oxide), hydroxyls, and CO can be found as marked with the grey areas in Figure S1(b). In the same manner we identify, in order of increasing binding energy, carbon atoms, hydrocarbons C_xH_y, and adsorbed CO in the C 1s spectra (see Figure S1(c)).

The adsorbed CO contribution in the O 1s spectrum could potentially overlap with a contribution caused by molecularly adsorbed H_2O. As the exact binding energy position of the adsorbed water might vary significantly depending on co-adsorbates, the oxidation state of the cobalt, and the amount of water[1,3,4], the CO and the H_2O contribution could not be easily distinguished during peak fitting. However, on clean Co(0001) as well as on O(ad)/Co(0001) in UHV at all investigated temperatures, it can be expected that water adsorbs dissociatively if it does so at all[4,5].

The atomic oxygen contribution to the O 1s spectra at low binding energies could stem from adsorbed atomic oxygen or from cobalt oxide. However, the Co 2p spectra show a mainly metallic surface in UHV (see Figure S1(a)). In a detailed fit (see below) merely 1 % and 6 % of the peak area are attributed to oxidized cobalt at 20 °C and 220 °C, respectively. With the calibration explained in the main text, the coverage of the oxygen can be estimated to 0.07 ML and 0.11 ML, respectively, which is thus partially caused by adsorbed atomic oxygen. Oxygen can be expected to easily form on the Co(0001) surface from water contamination[1,4], which is likely present in the UHV background, especially after the use of gases. The saturation coverage of 0.25 monolayers[6] is not reached in our case though. The amount of oxygen is increased at 220 °C, potentially because less surface area is covered by CO. As can be seen in Figure S2, the oxygen observed here is present until 300 °C. This suggests that the adsorbed oxygen might be stable under the annealing to 317 °C during the sample preparation. This is in agreement with temperature-programmed desorption experiments by Xu et al.[4], in which the adsorbed oxygen formed by water dissociation was stable until at least 350 °C. As CO was used in the flow cell previously, a significant amount of CO adsorbed from the UHV background can be found on this as-prepared sample in UHV at room temperature. Assuming the saturation coverage of 1/3 of a monolayer in UHV[7], we can use this measurement (with detailed fits as explained below) as a calibration to estimate coverages for the other adsorbed species (details see fitting section below).

Figure S2 shows estimated coverages on an as-prepared sample in UHV as a function of the surface temperature. These measurements were done at beamline 9.3.2[8] of the Advanced Light Source, Berkeley. Opposed to above measurements, these measurements were taken before any use of CO in the analysis chamber. Therefore, the amount of CO detected at room temperature is significantly lower and the amount of adsorbed oxygen does not show a specific temperature dependence staying between 0.1 ML and 0.13 ML. The CO is desorbed at 140 °C, which is in agreement with temperature-programmed desorption reported in the literature (see Figure S1 in Ref.[7]).

The atomic carbon coverage is estimated to be 0.08 ML (after previous use of CO in the chamber) at room temperature. As the annealing temperature of hcp cobalt is limited by a phase transition to fcc, the surface cannot be kept as flat as other surfaces and traces of contaminants, which preferably adsorb at lower-coordinated sites, are common (as for example also in Ref.[9]). However, we observe a reduced amount between 0.01 ML and 0.03 ML on different as-prepared samples measured at an increased temperature of 220 °C. In Figure S2 one can see that atomic C was not detectable anymore from 240 °C on. The desorption of carbon as CO can be excluded at all temperatures investigated here[10]. Removal of the carbon by reaction with background hydrogen can be expected from around 200 °C on as described in the main text. The dissolution of carbon in cobalt is only expected at significantly higher temperatures where cobalt carbide can be formed[11]. Theory suggests that carbon atoms can easily diffuse below the topmost surface layer of Co(0001), whereas they are unlikely to diffuse deeper into the bulk unless Co vacancies are present[12]. Carbon atoms right below the topmost surface layer should still be detectable in our case as we probe the two to three topmost layers of the cobalt crystal. However, the existence of vacancies is likely on a Co(0001) surface, such that the dissolution to lower layers cannot be excluded as an explanation for not detecting the carbon at these temperatures. In general, it is clear that keeping the surface at an elevated temperature (between 220 °C and 260 °C) in UHV and when intro-

[a] *Leiden Institute of Chemistry, Einsteinweg 55, 2333 CC Leiden, The Netherlands; Tel: +31 71 527 7361; E-mail: i.m.n.groot@lic.leidenuniv.nl*
[b] *Current Address: Peter Grünberg Institute (PGI-3), Forschungszentrum Jülich, 52425 Jülich, Germany*

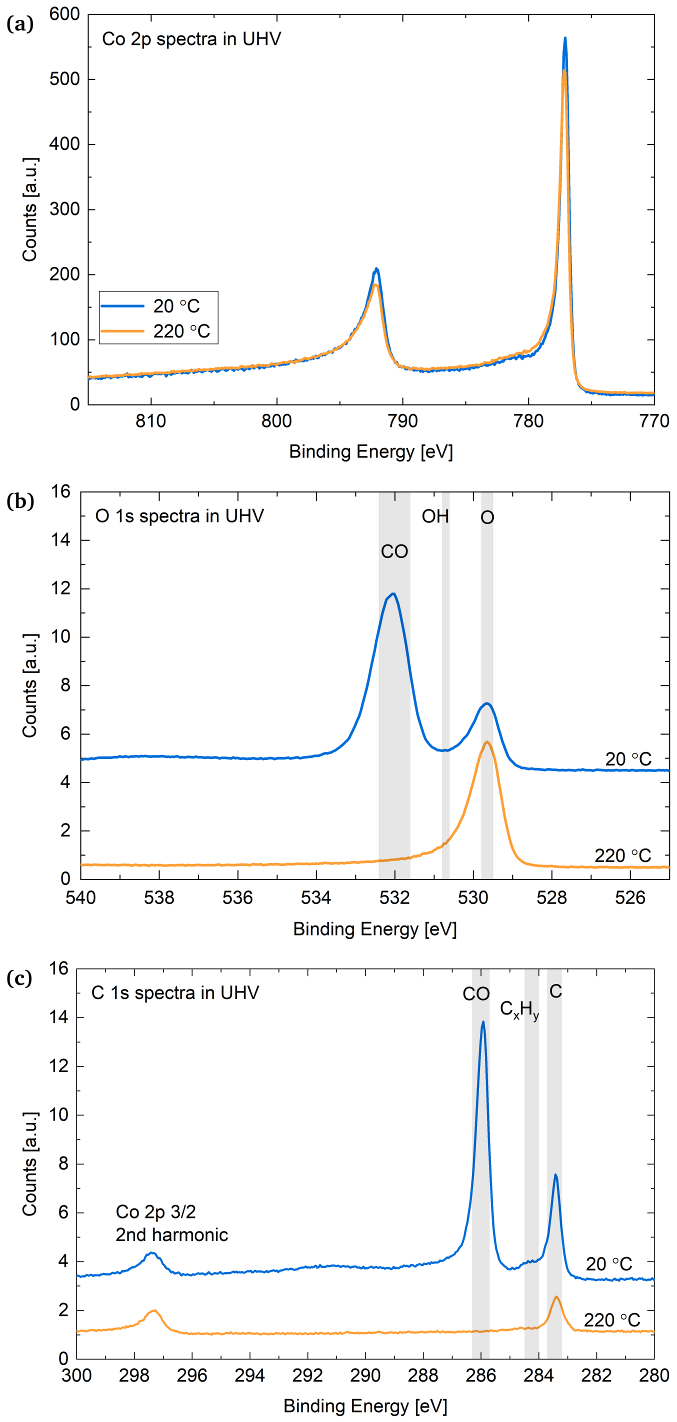

Fig. S1 (a) Co 2p, (b) O 1s, and (c) C 1s spectra measured on Co(0001) in UHV (base pressure on the order of 10^{-9} mbar with previous use of CO in the chamber) at 20 °C compared to 220 °C. For comparison the spectra are calibrated with respect to the cobalt signal as explained in detail in the text.

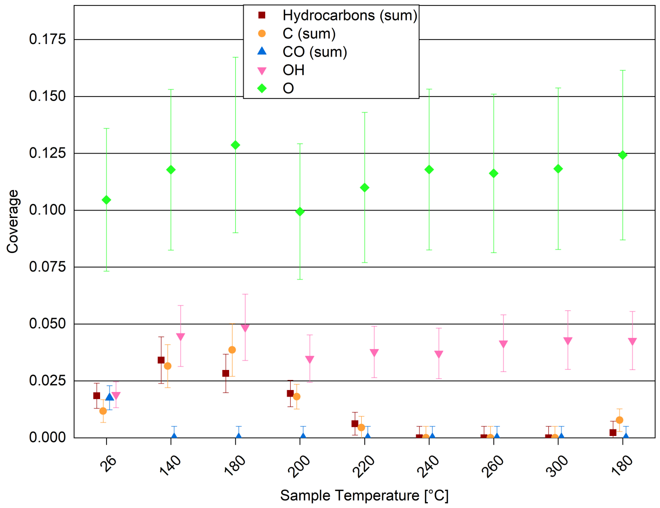

Fig. S2 Estimated coverages on an as-prepared Co(0001) in UHV (base pressure on the order of 10^{-9} mbar without previous use of CO in the chamber) at temperatures between 26 °C and 300 °C. As these measurements were taken at a different setup than the calibration measurement, the coverages reported are only a rough estimate based on a comparison to the amount of adsorbed atomic oxygen found in the measurements above in the same pressure range at 220 °C.

ducing gases can increase the surface cleanliness and keep the surface free from CO, carbon, and hydrocarbons.

Identification of the cobalt oxide present in H_2

Figure S3 shows a detailed fitting example of a Co 2p spectrum of (partially) oxidized Co(0001). The Co 2p peak was fitted with a main metallic peak at 778 eV of asymmetric shape LA(1,2.25,3). This is a pre-defined Lorentzian-type shape in CasaXPS [13], for which the low binding energy side of the Lorentzian is taken to the power of 2.25 and the resulting asymmetric shape is convoluted with a Gaussian of width 3). Additionally, two plasmon loss peaks of 30 % Lorentzian and 70 % Gaussian product line shape fixed at + 3 eV and + 5 eV and 6.4 % and 2.1 % peak area with respect to the main peak, respectively, are needed. This is based on the Co 2p fitting presented in Refs. [1,3] and adjusted until a satisfactory fit for a measurement on metallic cobalt is reached. Additionally, four oxidized peaks of 40 % Lorentzian and 60 % Gaussian product line shape at positions + 1.8 eV, + 4.1 eV, + 7.8 eV, and + 10 eV with respect to the metallic main peak are necessary. Although the distances between the different peaks caused by the oxide do not match exactly with those given in the literature, the spectra measured here are more similar to the spectrum of CoO (or $Co(OH)_2$) than to the spectrum of Co_3O_4 presented in Ref. [3]. Figure S3(b) shows a detailed fit of the O 1s spectrum of oxidized Co(0001) in wet H_2. The peaks are a 35 % Lorentzian and 65 % Gaussian product line shape at 530 eV and another at + 2.1 eV. As discussed in detail in the main text this suggests the presence of molecularly adsorbed water on top of the oxidized surface.

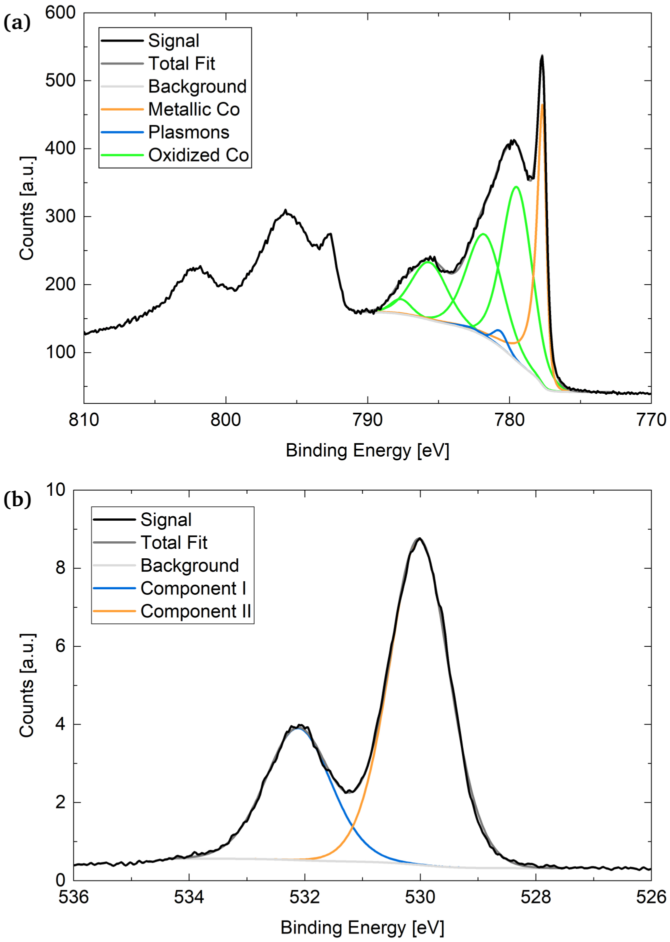

Fig. S3 (a) Co 2p spectrum measured on Co(0001) in 0.25 mbar (partially dried) H_2 at 220 °C surface temperature showing a fit of the 3/2 part of the doublet. This surface is in a partially oxidized state (caused by incomplete drying of the H_2 in the liquid nitrogen trap), which allows for an example fit clearly containing both metallic and oxidized cobalt. (b) O 1s spectrum measured on Co(0001) in wet H_2 at 220 °C including a fit. The spectrum corresponds to t = 11.6 min in Figure 1(a) of the main text.

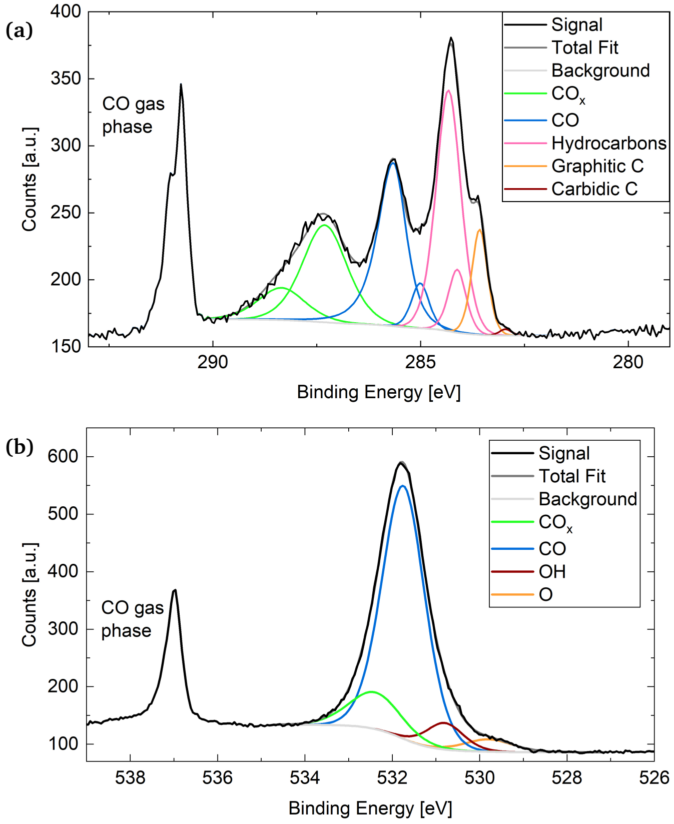

Fig. S5 (a) C 1s spectrum and (b) O 1s spectrum measured on Co(0001) in 0.25 mbar CO at (a) 190 °C and (b) 220 °C surface temperature showing the peaks necessary for a satisfactory fit.

Additional spectra of the metallic Co(0001) in 0.25 mbar CO

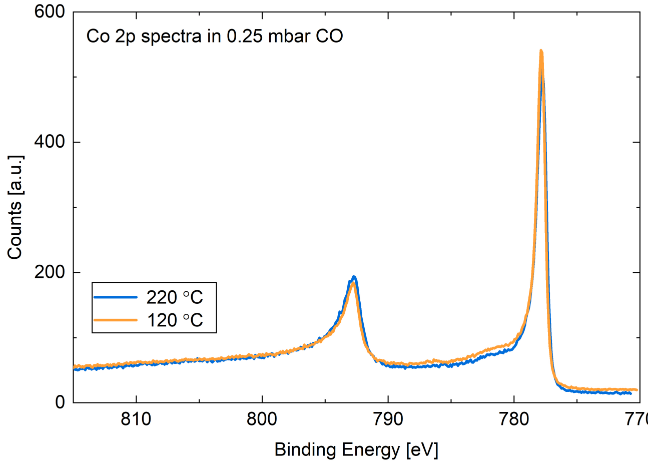

Fig. S4 Co 2p spectra measured on Co(0001) in 0.25 mbar CO at 220 °C and 120 °C.

Adsorbates: Fitting Procedure and Estimation of Coverages

In the main text, we identify the following adsorbates in the C 1s spectra: adsorbed CO_x, CO, hydrocarbons C_xH_y, and carbon atoms. To fit all C 1s spectra with the same set of peaks at fixed relative positions (see Figure S5(a) for an example), it is necessary to split the CO_x peak into two contributions. These are at + 2.69 eV and + 1.50 eV with respect to the main CO peak at 285.7 eV, which is best fitted with a slightly asymmetric LA(1.8,2.5,0) shape, for which the high binding-energy side of a Lorentzian shape is taken to the power of 1.8 and the other side to the power of 2.5. Additionally, a small contribution at - 0.65 eV from the main CO peak could stem from a different adsorption site on the surface [7,14] compared to the main CO peak. For a satisfactory fit, the hydrocarbon peak is split into two contributions as well, which have a distance of 0.2 eV from each other. In comparison with literature [15–17], the carbide/atomic carbon contribution is split into a graphitic carbon/multiple carbon atom contribution at 283.6 eV and a carbidic/single carbon atom contribution at - 0.37 eV with respect to the first. All C 1s peaks (except the main CO(ad) peak) were fitted satisfactorily with a 50 % Lorentzian and 50 % Gaussian product line shape. The relative binding energy positions of all contributions are fixed and the FWHM is restricted to the same value for every measurement of the same peak for consistency.

In the O 1s spectra, adsorbed CO_x, CO, OH, and O are identified in the main text. Figure S5(b) shows an example of the detailed fitting of the O 1s spectra with the atomic oxygen contribution at 529.7 eV (including a small contribution at 528 eV), the adsorbed hydroxyls at 530.7 eV, and the CO and CO_x peaks at 531.7 eV and 532.3 eV, respectively. The separation of the CO and CO_x peak is less clear in the O 1s spectra compared to the C 1s spectra and the absolute values of ratios between CO and CO_x peak areas are not clearly in agreement between the two. The disagreement could stem from the time that passes between measuring the C 1s and the O 1s spectra in every set of measurements. Additionally, the CO_x/CO ratio will be measured higher in the O 1s spectra whenever x is larger than 1. Therefore, the results discussed in the main text are based on the fitting of the C 1s spectra.

In order to estimate coverages from the detailed fits, the peak areas are calibrated for the slit used (see methods section in the main text) and the measured Co 2p area. Finally, the CO saturation coverage of 1/3 of a monolayer in UHV [7] was measured on the as-prepared sample in UHV (after the use of CO in the chamber, see Figure S1) and used as a calibration to estimate the coverages for the other adsorbed species. As non-negligible amounts of other adsorbed species are present in the same measurement, one needs to distinguish between the absolute CO coverage and the CO coverage on that part of the surface which is free from other adsorbates. We perform the calculation of the estimated absolute coverages in such a way that the CO coverage on the free part of the surface is equal to the saturation coverage which can correspond to a somewhat smaller absolute coverage. Using this calibration we find 0.02 ML coverage of hydrocarbons in UHV at room temperature, which likely stems from the increased CO background in the UHV after the use of CO in the high-pressure cell, and is decreased to below 0.01 ML when increasing the temperature as it likely desorbs.

Sulfur Coverages

Coverages of sulfur are roughly estimated by comparison with the calibration of the oxygen coverage by taking into account the different photon flux for the O 1s and the S 2p measurement as well as the cross sections of the respective orbitals with photons of the energy used (determined using Ref. [18]).

The sulfur coverage detected after a total of more than 17 h in 0.25 mbar CO is on the order of 0.001 ML.

Over the course of more than 7 h in 0.25 mbar H_2, a small amount of sulfur is adsorbed on the surface (0.007 ML in the last set of measurements at 300 °C), which likely stems from contaminants in the hydrogen bottle.

The estimated sulfur coverage increases to maximally 0.005 ML within 4 h in the reaction mixture for both gas ratios. Thus, although sulfur is known to easily block CO adsorption on Co(0001) [19,20] as well as on more industrially relevant catalysts [21], it is not a significant poison in the conditions and time frame studied here.

Beam Effect

Figure S6 compares the coverage of carbon and oxygen species measured over time in the reaction mixture at 220 °C while staying in one position on the surface (thus with strong beam exposure) to measurements where the beam exposure was minimized. To achieve the minimum beam exposure we have moved to a new position on the sample right before every measurement of the carbon binding energy range. In this way the influence by the beam is as small as possible and stays constant over time between measurements taken at the same temperature. When comparing two consecutive sweeps of every such carbon area measurement, no difference is detectable, which confirms that beam-induced deposition does not play a role in the time frame in which the carbon signal is measured. However, as the O 1s and Co 2p spectra are measured later in time, the areas could still be slightly influenced by a change in carbon species over time. The same procedure is used for all measurements shown in the main text and the previous sections of the supporting information.

In detail, the comparison in Figure S6 shows a somewhat higher coverage of C, likely stemming from beam-induced CO dissociation. Subtracting the two values at 2 h results in a maximum beam-induced C coverage of 0.06 ML. However, this difference decreases over time, likely because more surface area gets blocked by hydrocarbons on the surface with strong beam exposure. The detailed fits show that between 69 % and 80 % of the carbon is carbidic on the beam-exposed sample. This is in the same range as on the sample with minimized beam exposure (70 % to 76 %), thus suggesting that both carbide and graphite formation are promoted to roughly the same extent by the beam. The deposition of hydrocarbons is more strongly increased on the sample with strong beam exposure, roughly by a factor of 10 compared to the sample with minimum exposure. This suggests a beam-induced Fischer-Tropsch reaction. Figure S6(b) shows this

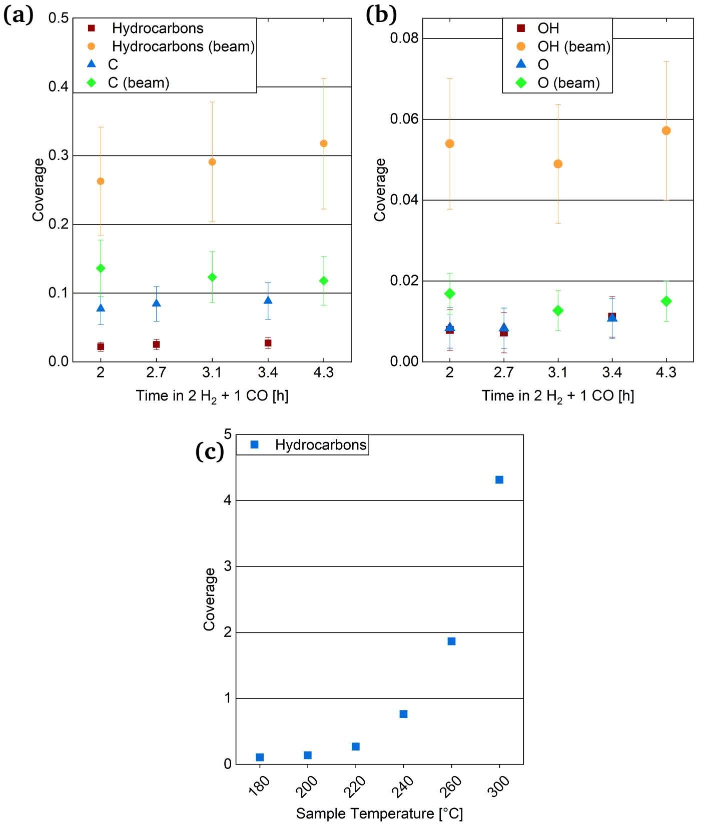

Fig. S6 Estimated coverages of (a) carbon species and (b) oxygen species measured on Co(0001) in 0.25 mbar 2 H_2 + 1 CO at 220 °C over time comparing measurements done with strong beam exposure, labeled (beam), to measurements done while minimizing beam exposure as explained in the text. (c) Hydrocarbon coverage on Co(0001) measured in 0.25 mbar 2 H_2 + 1 CO at increasing temperature. The sample was held at every temperature step for roughly 2.5 h under continuous beam exposure at the same position.

also leads to an increase in the estimated coverage of OH and slight increase in the coverage of O. Although this could stem from the water produced by the reaction, an oxidation of the surface has not been observed in any measurements with strong beam exposure.

As we also see a faster increase of hydrocarbon coverage when increasing the temperature in the measurements with minimal beam exposure (see Figure 10(b) in the main text), the beam might practically have a similar effect as an increased temperature. The beam-induced deposition could be observed starting from 220 °C surface temperature on and proceeds faster at higher temperatures. While increasing the temperature from 220 °C to 300 °C in the reaction mixture over a course of roughly 8 h, the hydrocarbon coverage increased exponentially (see Figure S6(b)) and reached a total of more than 4 layers estimated coverage. This additionally suggests that the cobalt surface itself might not be necessary for the beam-induced reaction, although the steps could still be involved. The hydrocarbons found on the surface do not seem to desorb in the temperature range investigated here, thus being able to cover the whole surface and making it crucial to minimize the beam exposure.

Author Contributions

S. Wenzel: main investigation, formal analysis, visualization, writing
D. Boden: investigation
R. van Lent: investigation
E. Motaee: investigation
M.K. Prabhu: investigation
H. Achour: investigation
I.M.N. Groot: conceptualization, supervision, funding acquisition

Conflicts of interest

There are no conflicts to declare.

Acknowledgements

This work was supported by the Dutch Research Council (NWO) [grant numbers 731.015.604 and 016.Vidi.189.022], BASF Nederland B.V., SABIC Global Technologies B.V., and Sasol Technology (Pty) Ltd. [grant number 731.015.604].

We acknowledge MAX IV Laboratory for time on Beamline HIPPIE under Proposal 20190786. Research conducted at MAX IV, a Swedish national user facility, is supported by the Swedish Research council under contract 2018-07152, the Swedish Governmental Agency for Innovation Systems under contract 2018-04969, and Formas under contract 2019-02496. Moreover, the research leading to this result has been supported by the project CALIPSOplus under the Grant Agreement 730872 from the EU Framework Program for Research and Innovation HORIZON 2020. We acknowledge local support at MAX IV by Andrey Shavorskiy, Mattia Scardamaglia, and Suyun Zhu.

This research used resources of the Advanced Light Source, a U.S. DOE Office of Science User Facility under contract no. DE-AC02-05CH11231. Part of the work presented was performed at beamline 9.3.2 at the Advanced Light Source under local support by Monika Blum, Yifan Ye, and Slavomir Nemsak.

References

[S1] C. H. Wu, B. Eren, H. Bluhm and M. B. Salmeron, *ACS Catalysis*, 2017, **7**, 1150–1157.

[S2] B. Böller, K. M. Durner and J. Wintterlin, *Nature Catalysis*, 2019, **2**, 1027–1034.

[S3] M. C. Biesinger, B. P. Payne, A. P. Grosvenor, L. W. Lau, A. R. Gerson and R. S. C. Smart, *Applied Surface Science*, 2011, **257**, 2717–2730.

[S4] L. Xu, Y. Ma, Y. Zhang, B. Chen, Z. Wu, Z. Jiang and W. Huang, *Journal of Physical Chemistry C*, 2010, **114**, 17023–17029.

[S5] W. Jiawei, J. Chen, Q. Guo, H. Y. Su, D. Dai and X. Yang, *Surface Science*, 2017, **663**, 56–61.

[S6] A. C. Kizilkaya, J. W. Niemantsverdriet and C. J. Weststrate, *Journal of Physical Chemistry C*, 2016, **120**, 4833–4842.

[S7] C. J. Weststrate, J. van de Loosdrecht and J. W. Niemantsverdriet, *Journal of Catalysis*, 2016, **342**, 1–16.

[S8] M. E. Grass, P. G. Karlsson, F. Aksoy, M. Lundqvist, B. Wannberg, B. S. Mun, Z. Hussain and Z. Liu, *Review of Scientific Instruments*, 2010, **81**, 053106.

[S9] P. Chai, H. Xu, L. Wu, H. Wang, C. Fu and W. Huang, *The Journal of Physical Chemistry C*, 2021, **125**, 22223–22230.

[S10] C. J. Weststrate, P. Van Helden, J. Van De Loosdrecht and J. W. Niemantsverdriet, *Surface Science*, 2016, **648**, 60–66.

[S11] D. Potoczna-Petru, *Carbon*, 1991, **29**, 73–79.

[S12] B. Li, Q. Zhang, L. Chen, P. Cui and X. Pan, *Physical Chemistry Chemical Physics*, 2010, **12**, 7848–7855.

[S13] `www.casaxps.com`, Casa Software Ltd.

[S14] J. Lahtinen, J. Vaari and K. Kauraala, *Surface Science*, 1998, **418**, 502–510.

[S15] C. J. Weststrate, A. C. Kizilkaya, E. T. Rossen, M. W. Verhoeven, I. M. Ciobîcă, A. M. Saib and J. W. Niemantsverdriet, *Journal of Physical Chemistry C*, 2012, **116**, 11575–11583.

[S16] B. Böller, M. Ehrensperger and J. Wintterlin, *ACS Catalysis*, 2015, **5**, 6802–6806.

[S17] L. Xu, Y. Ma, Z. Wu, B. Chen, Q. Yuan and W. Huang, *Journal of Physical Chemistry C*, 2012, **116**, 4167–4174.

[S18] `vuo.elettra.eu/services/elements/WebElements.html`, last acccessed on 29.03.2021.

[S19] K. Habermehl-Ćwirzeń and J. Lahtinen, *Surface Science*, 2004, **573**, 183–190.

[S20] M. Ehrensperger and J. Wintterlin, *Journal of Catalysis*, 2015, **329**, 49–56.

[S21] J. Barrientos, V. Montes, M. Boutonnet and S. Järås, *Catalysis Today*, 2016, **275**, 119–126.
